# Fatty acids and colorectal cancer: Insights from Mendelian randomization

**DOI:** 10.1097/MD.0000000000041768

**Published:** 2025-03-14

**Authors:** Dengge Jiang, Wenwen Yang, Yu Zhang

**Affiliations:** a Xi’AN No.1 Hospital, The First Affiliated Hospital of Northwest University, Xi’an, Shaanxi, China; b The First Clinical Medical College, Lanzhou University, Lanzhou, Gansu, China.

**Keywords:** colorectal cancer, docosahexaenoic acid, fatty acids, genetic variants, Mendelian randomization

## Abstract

Colorectal cancer (CRC) is one of the most common cancers worldwide, necessitating the identification of risk factors and preventive measures. Fatty acids, vital nutrients involved in various bodily functions, have been linked to CRC; however, findings are inconsistent. This Mendelian randomization study utilized data from the UK Biobank and included 18 fatty acid-related phenotypes. We used single-nucleotide polymorphisms as instrumental variables to examine the Causal connections between fatty acids and CRC. Statistical analysis involved the inverse-variance-weighted, Mendelian randomization-Egger, and weighted median methods to ensure robust findings. Our analysis revealed that docosahexaenoic acid and omega-3 fatty acids were positively associated with CRC risk. No significant associations were found between CRC and total fatty acids, saturated fatty acids, polyunsaturated fatty acids, or monounsaturated fatty acids. The degree of unsaturation was positively associated with CRC, while the ratio of omega-6 to omega-3 fatty acids was negatively associated. The study highlights a positive association between docosahexaenoic acid, omega-3 fatty acids, and CRC, suggesting that specific fatty acids may influence CRC risk. Further research in diverse populations is needed to confirm these findings and explore the underlying mechanisms.

## 1. Introduction

Globally, colorectal cancer (CRC) is the second most common cancer in women and the third most common in men.^[1]^ Considering the substantial burden of CRC worldwide, it is crucial to identify potential risk factors and develop preventive strategies to mitigate the disease. Fatty acids are essential nutrients in the body, serving as crucial components of cell membranes and playing significant roles in both health and disease.^[2]^ An increasing amount of research^[3–10]^ indicates that fatty acid intake is linked to CRC. However, these findings contradict previous Mendelian randomization (MR) studies.^[11,12]^ For example, while these studies suggested that increased intake of omega-3 fatty acids reduces the risk of CRC, two MR analyses^[11,12]^ found a positive relation between serum omega-3 fatty acids and CRC risk. MR is a type of statistical analysis employed in observational research to evaluate the relationships between different exposures and outcomes using genetic variants as instrumental variables (IVs). Genetic variants are randomly assigned during conception, which is the fundamental tenet of MR and is comparable to the randomization procedure in randomized controlled experiments.^[13]^ Our study utilized 18 fatty acid-related phenotypes from the UK Biobank to analyze the impact of fatty acids on CRC, providing additional evidence to elucidate their relationship.

## 2. Method

The following premises underpin the MR study: relevance assumption – there is a strong association between the exposure and the IVs; independence assumption – confounding factors do not influence the IVs; exclusion restriction assumption – the IVs impact the outcome solely through their effect on the exposure, without other indirect pathways.

### 2.1. Data sources

Our investigation included 18 fatty acids as exposures, sourced from the UK Biobank.^[14]^ We categorized these eighteen fatty acids into 5 groups: total, saturated, unsaturated, omega-3, and omega-6. These fatty acids encompassed both absolute levels and relative proportions (such as the proportion of linoleic acid to total fatty acids). More information about these fatty acids is provided in Table 1. The outcome of this study, CRC, included 19,948 cases and 12,124 controls, sourced from a publicly published study^[15]^ by the OpenGwas project. Ethical review is not necessary because the data used in this study have been anonymized and are accessible to the public.

**Table 1:** Information on exposure and outcome datasets.

Exposure/outcome	Classification	Exposure or outcome names	Phenocode in IEU OpenGWAS project	Sample size	The article or biobank of the data source	Number of cases	Number of controls	URL in the UK Biobank
Exposure	Total fatty acid	Total fatty acid levels	id:ebi-a-GCST90092987	115,006	PMID: 35213538	N/A	N/A	https://biobank.ctsu.ox.ac.uk/crystal/field.cgi?id=23442
	Saturated fatty acid	Saturated fatty acid levels	id:ebi-a-GCST90092980	115,006	PMID: 35213538	N/A	N/A	https://biobank.ctsu.ox.ac.uk/crystal/field.cgi?id=23448
		Ratio of saturated fatty acids to total fatty acids	id:ebi-a-GCST90092981	115,006	PMID: 35213538	N/A	N/A	https://biobank.ctsu.ox.ac.uk/crystal/field.cgi?id=23455
	Unsaturated fatty acid	Degree of unsaturation	id:ebi-a-GCST90092994	115,006	PMID: 35213538	N/A	N/A	https://biobank.ctsu.ox.ac.uk/crystal/field.cgi?id=23443
		Ratio of polyunsaturated fatty acids to total fatty acids	id:ebi-a-GCST90092941	115,006	PMID: 35213538	N/A	N/A	https://biobank.ctsu.ox.ac.uk/crystal/field.cgi?id=23453
		Ratio of polyunsaturated fatty acids to monounsaturated fatty acids	id:ebi-a-GCST90092940	115,006	PMID: 35213538	N/A	N/A	https://biobank.ctsu.ox.ac.uk/crystal/field.cgi?id=23458
		Polyunsaturated fatty acid levels	id:ebi-a-GCST90092939	115,006	PMID: 35213538	N/A	N/A	https://biobank.ctsu.ox.ac.uk/crystal/field.cgi?id=23446
		Ratio of monounsaturated fatty acids to total fatty acids	id:ebi-a-GCST90092929	115,006	PMID: 35213538	N/A	N/A	https://biobank.ctsu.ox.ac.uk/crystal/field.cgi?id=23454
		Monounsaturated fatty acid levels	id:ebi-a-GCST90092928	115,006	PMID: 35213538	N/A	N/A	https://biobank.ctsu.ox.ac.uk/crystal/field.cgi?id=23447
	Omega-3 fatty acid	Ratio of omega-3 fatty acids to total fatty acids	id:ebi-a-GCST90092932	115,006	PMID: 35213538	N/A	N/A	https://biobank.ctsu.ox.ac.uk/crystal/field.cgi?id=23451
		Ratio of docosahexaenoic acid to total fatty acid levels	id:ebi-a-GCST90092817	115,006	PMID: 35213538	N/A	N/A	https://biobank.ctsu.ox.ac.uk/crystal/field.cgi?id=23457
		Omega-3 fatty acid levels	id:ebi-a-GCST90092931	115,006	PMID: 35213538	N/A	N/A	https://biobank.ctsu.ox.ac.uk/crystal/field.cgi?id=23444
		Docosahexaenoic acid levels	id:ebi-a-GCST90092816	115,006	PMID: 35213538	N/A	N/A	https://biobank.ctsu.ox.ac.uk/crystal/field.cgi?id=23450
	Omega-6 fatty acid	Ratio of omega-6 fatty acids to total fatty acids	id:ebi-a-GCST90092935	115,006	PMID: 35213538	N/A	N/A	https://biobank.ctsu.ox.ac.uk/crystal/field.cgi?id=23452
		Ratio of omega-6 fatty acids to omega-3 fatty acids	id:ebi-a-GCST90092934	115,006	PMID: 35213538	N/A	N/A	https://biobank.ctsu.ox.ac.uk/crystal/field.cgi?id=23459
		Ratio of linoleic acid to total fatty acids	id:ebi-a-GCST90092881	115,006	PMID: 35213538	N/A	N/A	https://biobank.ctsu.ox.ac.uk/crystal/field.cgi?id=23456
		Omega-6 fatty acid levels	id:ebi-a-GCST90092933	115,006	PMID: 35213538	N/A	N/A	https://biobank.ctsu.ox.ac.uk/crystal/field.cgi?id=23445
		Linoleic acid levels	id:ebi-a-GCST90092880	115,006	PMID: 35213538	N/A	N/A	https://biobank.ctsu.ox.ac.uk/crystal/field.cgi?id=23449
Outcome	N/A	Colorectal cancer	ebi-a-GCST012879	32,072	PMID:30510241	19,948	12,124	N/A

In this table, we presented information such as the names, sources, and sample sizes of fatty acids and colorectal cancer. More information about exposure and outcomes is available at the IEU OpenGWAS project (https://gwas.mrcieu.ac.uk/).

GWAS = Genome-Wide Association Studies, IEU = integrative epidemiology unit, NA and N/A: = not applicable, SNPs = single nucleotide polymorphisms.

### 2.2. Selection of IVs

Researchers were able to delve deeper into possible causal links between the exposures and the outcome in the MR analysis by employing single-nucleotide polymorphisms (SNPs) as IVs. The criteria employed to identify SNPs for our research included a clumping window of 10,000 kb, strong linkage disequilibrium (r2 < 0.001), and significance below the genome-wide threshold (*P* < 5 × 10^–8^). SNPs that exhibited a significant correlation with the outcome (*P* < 5 × 10–8) were eliminated from each analysis to minimize bias and comply with MR assumptions.

### 2.3. Statistical analysis

Three well-known MR analysis techniques were applied in our research to assess causal associations: the inverse-variance-weighted (IVW) method, chosen as the primary technique due to its superior ability to determine causal relationships compared to the other 2 methods – the MR-Egger technique and the weighted median (WM) technique. Although the IVW method is the most effective, it must meet certain conditions: horizontal pleiotropy is required to remain balanced or negligible.^[16]^ Applying false discovery rate^[17]^ corrections to the *P*-values derived from the IVW technique can mitigate the false discovery rate during multiple hypothesis testing procedures. We detected outliers through the application of the MR-pleiotropy residual sum and outlier technique. We employed Cochran’s Q-test for measuring heterogeneity. The fixed-effects IVW technique was adopted in cases where heterogeneity was not present (*P* > .05), and the random-effects IVW technique was adopted when it was. For all statistical analyses, the TwoSampleMR package^[18]^ (version 0.5.10) in R (version 4.3.2) was used.

## 3. Result

Our study incorporated absolute and relative levels of a total of 18 fatty acids, employing MR analysis to investigate their impact on CRC. This study utilized CRC as the outcome, involving 19,948 individuals and 12,124 controls from a publicly published study.^[15]^ We examined the effects of eighteen fatty acids on CRC, excluding SNPs linked to CRC. Table S1, Supplemental Digital Content, http://links.lww.com/MD/O493 displays the results of the MR-pleiotropy residual sum and outlier technique along with the 3 MR analysis techniques. Subsequently, we removed detected outliers, repeated all analyses, and presented the results in Table 2. In our study, no horizontal pleiotropy was detected. As the most effective of the 3 methods for detecting causal links, our research focuses mainly on the IVW technique’s analytical results. It is noteworthy that all 3 MR analysis methods yielded consistent conclusions: docosahexaenoic acid (DHA) and omega-3 fatty acid are risk factors for CRC.

**Table 2:** The results of the three Mendelian randomization method on the impact of fatty acids on colorectal cancer after removing outliers.

	Outcome	Exposure	Number of SNPs	IVW_Beta	IVW_se	IVW_pval	IVW_q_value	Weighted_median_Beta	Weighted_median_se	Weighted_median_pval	MR_Egger_Beta	MR_Egger_se	MR_Egger_pval	Q	Q_pval	egger_intercept	se	pval
Total fatty acid	Colorectal cancer ‖ id:ebi-a-GCST012879	Total fatty acid levels ‖ id:ebi-a-GCST90092987	55	4.33E-02	6.77E-02	5.22E-01	7.97E-01	8.35E-02	8.45E-02	3.23E-01	6.97E-02	1.20E-01	5.65E-01	9.45E + 01	5.38E-04	−1.84E-03	6.91E-03	7.91E-01
Saturated fatty acid	Colorectal cancer ‖ id:ebi-a-GCST012879	Saturated fatty acid levels ‖ id:ebi-a-GCST90092980	47	3.83E-02	6.87E-02	5.77E-01	7.99E-01	9.55E-02	9.34E-02	3.07E-01	7.74E-02	1.26E-01	5.41E-01	6.61E + 01	2.75E-02	−2.60E-03	6.98E-03	7.11E-01
	Colorectal cancer ‖ id:ebi-a-GCST012879	Ratio of saturated fatty acids to total fatty acids ‖ id:ebi-a-GCST90092981	24	−5.29E-02	1.15E-01	6.47E-01	8.32E-01	−1.36E-03	1.66E-01	9.93E-01	−2.14E-01	2.85E-01	4.61E-01	3.13E + 01	1.16E-01	8.31E-03	1.34E-02	5.42E-01
Unsaturated fatty acid	Colorectal cancer ‖ id:ebi-a-GCST012879	Degree of unsaturation ‖ id:ebi-a-GCST90092994	38	1.69E-01	5.53E-02	2.20E-03	6.61E-03	2.24E-01	5.26E-02	2.06E-05	2.09E-01	7.34E-02	7.24E-03	5.81E + 01	1.48E-02	−5.16E-03	6.24E-03	4.14E-01
	Colorectal cancer ‖ id:ebi-a-GCST012879	Ratio of polyunsaturated fatty acids to total fatty acids ‖ id:ebi-a-GCST90092941	42	−1.82E-02	7.68E-02	8.12E-01	8.55E-01	−5.92E-02	1.08E-01	5.84E-01	8.28E-03	1.41E-01	9.54E-01	5.02E + 01	1.54E-01	−1.60E-03	7.11E-03	8.23E-01
	Colorectal cancer ‖ id:ebi-a-GCST012879	Ratio of polyunsaturated fatty acids to monounsaturated fatty acids ‖ id:ebi-a-GCST90092940	48	1.27E-02	6.70E-02	8.50E-01	8.55E-01	−2.79E-02	9.35E-02	7.65E-01	−8.56E-02	1.12E-01	4.48E-01	6.30E + 01	5.90E-02	6.95E-03	6.35E-03	2.79E-01
	Colorectal cancer ‖ id:ebi-a-GCST012879	Polyunsaturated fatty acid levels ‖ id:ebi-a-GCST90092939	50	3.83E-02	6.11E-02	5.31E-01	7.97E-01	9.65E-02	7.70E-02	2.10E-01	1.26E-01	1.19E-01	2.93E-01	6.91E + 01	3.07E-02	−6.16E-03	7.11E-03	3.91E-01
	Colorectal cancer ‖ id:ebi-a-GCST012879	Ratio of monounsaturated fatty acids to total fatty acids ‖ id:ebi-a-GCST90092929	53	1.09E-02	5.97E-02	8.55E-01	8.55E-01	4.50E-02	8.48E-02	5.96E-01	5.96E-02	9.67E-02	5.41E-01	6.43E + 01	1.17E-01	−3.57E-03	5.57E-03	5.24E-01
	Colorectal cancer ‖ id:ebi-a-GCST012879	Monounsaturated fatty acid levels ‖ id:ebi-a-GCST90092928	63	6.52E-02	5.79E-02	2.60E-01	5.84E-01	6.36E-02	8.58E-02	4.59E-01	6.31E-02	9.61E-02	5.14E-01	8.50E + 01	2.80E-02	1.54E-04	5.43E-03	9.77E-01
Omega-3 fatty acid	Colorectal cancer ‖ id:ebi-a-GCST012879	Ratio of omega-3 fatty acids to total fatty acids ‖ id:ebi-a-GCST90092932	32	1.64E-01	4.54E-02	2.97E-04	1.34E-03	1.96E-01	4.66E-02	2.50E-05	2.21E-01	5.75E-02	5.85E-04	4.12E + 01	1.03E-01	−8.81E-03	5.66E-03	1.30E-01
	Colorectal cancer ‖ id:ebi-a-GCST012879	Ratio of docosahexaenoic acid to total fatty acid levels ‖ id:ebi-a-GCST90092817	24	2.13E-01	6.88E-02	1.96E-03	6.61E-03	2.76E-01	6.31E-02	1.21E-05	2.91E-01	9.76E-02	6.83E-03	3.73E + 01	3.02E-02	−9.43E-03	8.37E-03	2.72E-01
	Colorectal cancer ‖ id:ebi-a-GCST012879	Omega-3 fatty acid levels ‖ id:ebi-a-GCST90092931	47	1.74E-01	4.22E-02	3.63E-05	3.27E-04	2.25E-01	5.10E-02	1.03E-05	2.20E-01	6.00E-02	6.37E-04	5.36E + 01	2.07E-01	−5.70E-03	5.28E-03	2.86E-01
	Colorectal cancer ‖ id:ebi-a-GCST012879	Docosahexaenoic acid levels ‖ id:ebi-a-GCST90092816	39	2.18E-01	5.20E-02	2.72E-05	3.27E-04	2.66E-01	6.03E-02	1.05E-05	2.90E-01	7.43E-02	3.84E-04	4.58E + 01	1.79E-01	−7.68E-03	5.71E-03	1.87E-01
Omega-6 fatty acid	Colorectal cancer ‖ id:ebi-a-GCST012879	Ratio of omega-6 fatty acids to total fatty acids ‖ id:ebi-a-GCST90092935	46	−5.53E-02	7.17E-02	4.40E-01	7.93E-01	−8.56E-02	9.85E-02	3.85E-01	2.82E-02	1.23E-01	8.20E-01	6.10E + 01	5.62E-02	−5.66E-03	6.79E-03	4.09E-01
	Colorectal cancer ‖ id:ebi-a-GCST012879	Ratio of omega-6 fatty acids to omega-3 fatty acids ‖ id:ebi-a-GCST90092934	28	−1.75E-01	4.45E-02	8.22E-05	4.93E-04	−2.07E-01	4.58E-02	6.56E-06	−2.42E-01	5.96E-02	4.04E-04	3.31E + 01	1.92E-01	1.02E-02	6.30E-03	1.17E-01
	Colorectal cancer ‖ id:ebi-a-GCST012879	Ratio of linoleic acid to total fatty acids ‖ id:ebi-a-GCST90092881	31	−2.34E-01	1.29E-01	6.94E-02	1.78E-01	−2.10E-01	1.68E-01	2.11E-01	−1.91E-01	3.21E-01	5.57E-01	4.02E + 01	1.00E-01	−1.70E-03	1.16E-02	8.85E-01
	Colorectal cancer ‖ id:ebi-a-GCST012879	Omega-6 fatty acid levels ‖ id:ebi-a-GCST90092933	52	2.10E-02	6.43E-02	7.44E-01	8.55E-01	1.05E-01	8.79E-02	2.33E-01	2.12E-01	1.24E-01	9.44E-02	7.00E + 01	3.97E-02	−1.24E-02	6.97E-03	8.08E-02
	Colorectal cancer ‖ id:ebi-a-GCST012879	Linoleic acid levels ‖ id:ebi-a-GCST90092880	42	6.17E-02	6.08E-02	3.10E-01	6.20E-01	1.04E-01	9.16E-02	2.55E-01	2.80E-01	1.24E-01	2.92E-02	4.34E + 01	3.71E-01	−1.41E-02	7.01E-03	5.15E-02

The impact of fatty acids on colorectal cancer after removing outliers. We present the outcomes of three Mendelian randomization analyses. The IVW method, known for its heightened sensitivity in detecting causality, serves as our primary tool to ascertain the presence of a causal relationship. The MR-Egger method is also employed for detecting horizontal pleiotropy due to its allowance for the presence of nonzero intercepts (The columns in the table corresponding to “egger_intercept,” “se,” and “pval”), a pval below 0.05 suggests the existence of horizontal pleiotropy. Heterogeneity was assessed using Cochran’s Q test (The columns in the table corresponding to “Q” and “Q_pval”).

IVW = inverse variance weighted method, lci95 = lower 95% confidence interval, MR_Egger = MR-Egger method, q_value = the *P* value post FDR method (Benjamini and Hochberg) corrected, SNPs = single-nucleotide polymorphisms, uci95 = upper 95% confidence interval, Weighted_median = weighted median method.

### 3.1. Total fatty acid levels and CRC

We observed no association between total fatty acid levels and CRC across 3 different methods: IVW (β = 0.0433, *P* = .522), WM (β = 0.0835, *P* = .323), and MR-Egger (β = 0.0697, *P* = .565). Additional information is presented in Table 2.

### 3.2. Saturated fatty acids and CRC

We found no significant association between saturated fatty acids, both in absolute levels (IVW: β = 0.0383, *P* = .577; WM: β = 0.0955, *P* = .307; MR-Egger: β = 0.0774, *P* = .541) and as a ratio to total fatty acids (IVW: β = −0.0529, *P* = .647; WM: β = −0.00136, *P* = .993; MR-Egger: β = −0.214, *P* = .461), and CRC. Additional information is presented in Table 2.

### 3.3. Unsaturated fatty acids and CRC

We analyzed the degree of unsaturation, as well as the absolute and relative levels of polyunsaturated fatty acids (PUFAs) and monounsaturated fatty acids (MUFAs), to assess their impact on CRC. Our findings indicated that only the degree of unsaturation (IVW: β = 0.169, *P* = .00220; WM: β = 0.224, *P* < .0001; MR-Egger: β = 0.209, *P* = .00724) was positively associated with CRC. No significant association was found between PUFAs, both in absolute levels (IVW: β = 0.0383, *P* = .531; WM: β = 0.0965, *P* = .210; MR-Egger: β = 0.126, *P* = .293) and as a ratio to total fatty acids (IVW: β = −0.0182, *P* = .812; WM: β = −0.0592, *P* = .584; MR-Egger: β = 0.00828, *P* = .954), and CRC. Similarly, no significant association was observed between MUFAs, both in absolute levels (IVW: β = 0.0652, *P* = .260; WM: β = 0.0636, *P* = .459; MR-Egger: β = 0.0631, *P* = .514) and as a ratio to total fatty acids (IVW: β = 0.0109, *P* = .855; WM: β = 0.0450, *P* = .596; MR-Egger: β = 0.0596, *P* = .541), and CRC. Our study also found that the ratio of PUFAs to MUFAs (IVW: β = 0.0127, *P* = .850; WM: β = −0.0279, *P* = .765; MR-Egger: β = −0.0856, *P* = .448) was not associated with CRC. Additional information is presented in Table 2.

### 3.4. Omega-3 fatty acids and CRC

We observed a positive correlation between DHA and CRC, both in absolute levels (IVW: β = 0.218, *P* < .0001; WM: β = 0.266, *P* < .0001; MR-Egger: β = 0.290, *P* = .000384) and as a ratio to total fatty acids (IVW: β = 0.213, *P* = .00196; WM: β = 0.276, *P* < .0001; MR-Egger: β = 0.291, *P* = .00683). Similarly, we found a positive correlation between omega-3 fatty acids and CRC, both in absolute levels (IVW: β = 0.174, *P* < .0001; WM: β = 0.225, *P* < .0001; MR-Egger: β = 0.220, *P* = .000637) and as a ratio to total fatty acids (IVW: β = 0.164, *P* = .000297; WM: β = 0.196, *P* < .0001; MR-Egger: β = 0.221, *P* = .000585). Additional information is presented in Table 2.

### 3.5. Omega-6 fatty acids and CRC

We observed almost no significant association between linoleic acid and CRC, both in absolute levels (IVW: β = 0.0617, *P* = .310; WM: β = 0.104, *P* = .255; MR-Egger: β = 0.280, *P* = .0292) and as a ratio to total fatty acids (IVW: β = −0.234, *P* = .0694; WM: β = −0.210, *P* = .211; MR-Egger: β = −0.191, *P* = .557). Similarly, no significant association was observed between omega-6 fatty acids and CRC, both in absolute levels (IVW: β = 0.0210, *P* = .744; WM: β = 0.105, *P* = .233; MR-Egger: β = 0.212, *P* = .0944) and as a ratio to total fatty acids (IVW: β = −0.0553, *P* = .440; WM: β = −0.0856, *P* = .385; MR-Egger: β = 0.0282, *P* = .820). We did observe a negative correlation between the ratio of omega-6 fatty acids to omega-3 fatty acids and CRC (IVW: β = −0.175, *P* < .0001; WM: β = −0.207, *P* < .0001; MR-Egger: β = −0.242, *P* = .000404). Additional information is presented in Table 2.

## 4. Discussion

Our study included a total of 18 fatty acid-related indices, primarily comprising the absolute or relative levels of saturated fatty acids, omega-3 fatty acids, unsaturated fatty acids, and omega-6 fatty acids, and analyzed their impact on CRC. Although several prior MR studies^[11,12]^ have investigated the relationship between circulating PUFAs and CRC, they included relatively small sample sizes^[19,20]^ and fewer SNPs.^[11,12]^ In contrast, our study utilized serum fatty acids from over 110,000 participants in the UK Biobank,^[14]^ enabling us to identify a greater number of eligible IVs. Our study indicated that only the degree of unsaturation in the blood was positively linked to CRC, while total fatty acids, saturated fatty acids, PUFAs, and MUFAs were not associated with the risk of CRC. Research^[21]^ has found that the degree of unsaturation of fatty acids in plasma exosomes of CRC patients can serve as a potential biomarker. A prospective study from the UK^[22]^ did not find any association between saturated fat intake, total fat intake, and unsaturated fat intake with CRC. A study from China^[22]^ also found no significant association between saturated fatty acids, MUFAs, and PUFAs intake and CRC risk. We found that both DHA and omega-3 fatty acids were positively linked to CRC, whether in absolute levels or relative to total fatty acids. Omega-3 fatty acids are a class of PUFAs, primarily including alpha-linolenic acid, eicosapentaenoic acid (EPA), and DHA.^[23]^ Due to our study’s exclusive focus on DHA and the absence of data on the other 2 omega-3 fatty acids, we cannot determine whether the relation between omega-3 fatty acids and CRC is primarily attributable to the effects of DHA alone or involves interactions among various omega-3 fatty acids. Numerous epidemiological studies^[3–10]^ have indicated that a higher intake of omega-3 fatty acids, such as EPA and DHA, can reduce the risk of CRC. However, evidence from MR studies^[11,12]^ suggested a positive correlation between omega-3 fatty acids and the risk of CRC. Our study utilized data different from previous MR studies to validate the conclusion that omega-3 fatty acids enhance the risk of CRC. Several studies^[24–27]^ have found that CRC tissue contains significantly higher levels of omega-3 PUFAs compared to adjacent normal tissue. This further highlights the necessity for future research to clarify the relationship between omega-3 fatty acids and CRC. This study also found that the absolute and relative levels of linoleic acid and omega-6 fatty acids are unrelated to CRC, while the proportion of omega-6 fatty acids to omega-3 fatty acids is negatively associated with CRC. Given the positive correlation between omega-3 fatty acids and CRC, the negative correlation between the proportion of omega-6 fatty acids to omega-3 fatty acids and CRC is likely attributable to the impact of omega-3 fatty acids. Therefore, our study primarily reveals a positive relation between DHA and omega-3 fatty acids with CRC, while other fatty acids are unrelated to CRC.

There is no denying the inherent limitations of this research. Firstly, we cannot determine whether the relationship between omega-3 fatty acids and CRC is primarily due to the effects of DHA alone or involves interactions among different omega-3 fatty acids because our study focused exclusively on DHA and lacked data on the other omega-3 fatty acids. Secondly, the applicability of our findings to other populations is uncertain due to the predominance of exposure and outcome data collected from European populations. This limitation underscores the necessity for further research to validate our results in larger and more diverse populations, casting doubt on their relevance to ethnically or geographically diverse groups. Thirdly, the absence of summary-level Genome-Wide Association Study data stratified by age and gender impedes the implementation of stratified analyses based on these variables.

## 5. Conclusion

Our comprehensive study demonstrated a positive linkage between DHA and omega-3 fatty acids with the risk of CRC. However, no significant associations were found between CRC and total fatty acids, saturated fatty acids, PUFAs, or MUFAs. Further research involving diverse populations and additional omega-3 fatty acids is necessary to elucidate the mechanisms underlying these associations and to confirm the generalizability of our findings.

## Acknowledgments

Special thanks to the UK Biobank, and the IEU open GWAS project developed by The MRC Integrative Epidemiology Unit (IEU) at the University of Bristol.

## Author contributions

**Conceptualization:** Dengge Jiang, Wenwen Yang, Yu Zhang.

**Data curation:** Dengge Jiang, Wenwen Yang, Yu Zhang.

**Formal analysis:** Dengge Jiang, Wenwen Yang, Yu Zhang.

**Funding acquisition:** Dengge Jiang, Wenwen Yang, Yu Zhang.

**Investigation:** Dengge Jiang, Wenwen Yang, Yu Zhang.

**Methodology:** Dengge Jiang, Wenwen Yang, Yu Zhang.

**Project administration:** Dengge Jiang, Wenwen Yang, Yu Zhang.

**Resources:** Dengge Jiang, Wenwen Yang, Yu Zhang.

**Software:** Dengge Jiang, Wenwen Yang, Yu Zhang.

**Supervision:** Dengge Jiang, Wenwen Yang, Yu Zhang.

**Validation:** Dengge Jiang, Wenwen Yang, Yu Zhang.

**Visualization:** Dengge Jiang, Wenwen Yang, Yu Zhang.

**Writing – original draft:** Dengge Jiang, Yu Zhang.

**Writing – review & editing:** Yu Zhang.

## Supplementary Material


